# Novel compound heterozygous mutation in *WEE2* is associated with fertilization failure: case report of an infertile woman and literature review

**DOI:** 10.1186/s12905-020-01111-5

**Published:** 2020-11-04

**Authors:** Ye Tian, Guojie Wang, Jin Wang, Xiaohuan Mu, Haixia Chen, Xueru Song, Xiaohong Bai

**Affiliations:** Reproductive Medicine Center, Department of Gynecology and Obstetrics, Tianjin Medical University General Hospital, 154, Anshan Road, Heping District, Tianjin, 300052 China

**Keywords:** Artificial oocyte activation, Human fertilization failure, Novel variants, *WEE2*

## Abstract

**Background:**

Fertilization failure after intracytoplasmic sperm injection continues to affect couples and the etiology is not well-understood.

**Case presentation:**

We characterized a couple with 2-year history of primary unexplained infertility. Three different assisted reproduction attempts (IVF + rescue ICSI, ICSI and ICSI-AOA) showed repeated fertilization failure for MII oocyte retrieval after controlled ovarian hyperstimulation. After whole-exome sequencing and sanger sequencing of the couple and their family members, variant pathogenicity was assessed using SIFT, PolyPhen2, Mutation Taster, and Human Splicing Finder software. We identified novel compound heterozygous mutations, c.1535 + 3A > G and c.946C > T (p. Leu316Phe), in *WEE2* in the female proband. Trios analysis of the variations revealed an autosomal recessive pattern. c.1535 + 3A > G in *WEE2* was predicted to break the wild-type donor site and affect splicing, and the missense mutation c.946C > T (p. Leu316Phe) of WEE2 was predicted to be pathogenic.

**Conclusion:**

A novel compound heterozygous mutation in *WEE2* was identified in an infertile female who experienced repeated fertilization failure even after ICSI-AOA. These novel mutations in *WEE2* provided genetic evidence for fertilization failure.

## Background

Total fertilization failure (TFF) occurs in 5–10% of total in vitro fertilization (IVF) cycles [1]. TFF is a mentally and financially devasting event for infertile couples. Since 1992, intracytoplasmic sperm injection (ICSI) has been widely used to improve fertilization rates in couples with male factor infertility. However, TFF still occurs in 1–3% of ICSI cycles [2].

The etiology underlying TFF remains unclear. Several studies have shown that oocyte activation defects are the main cause of fertilization failure after ICSI. Oocyte activation is a complex process initiated by intracellular calcium oscillations after sperm enter the ooplasm. Artificial oocyte activation (AOA) is a technique that triggers an artificial increase in intracellular calcium. ICSI followed by AOA (ICSI-AOA) is typically used to improve the fertilization rate of patients with male factor-related oocyte activation defects [3]. Genetic factors play important roles in oocyte activation defects and likely result in repeated human fertilization failure even after ICSI-AOA. Given the importance of oocyte- and sperm-related factors in oocyte activation, deleterious mutations in oocyte-specific genes and sperm-specific genes can result in TFF in humans. A mutation in the sperm-specific gene *PLCZ1* was showed to cause abnormal calcium oscillations and TFF in two infertile brothers with normal sperm morphology [4]. A mutation in the maternal effect gene *TLE6* impaired oocyte meiosis and led to a low fertilization rate and early cleavage failure in two families with female infertility [5]. Recently, Sang et al. [6] identified a mutation in the oocyte-specific gene *WEE2* which resulted in the failure of pronuclei formation and human fertilization in four independent infertile women.

WEE2 (WEE1 homolog 2, also known as WEE1B) belongs to the WEE kinase family and is conserved from yeast to humans [7]. A previous study indicated that in mouse oocytes at the germinal vesicle stage, Wee2 acted as a key maturation-promoting factor inhibitory kinase and is involved in maintaining meiotic arrest [8]. In mouse oocytes at metaphase II stage, Wee2 inhibited the phosphorylation of Cdc2, which was required for metaphase II exit. As a result, downregulation of Wee2 during egg activation leads to failure of pronucleus formation even after calcium oscillations [9]. Recently, mutations in *WEE2* were found to be associated with human pronucleus formation and repeated fertilization failure [6, 10–14]. In this study, whole-exome sequencing was performed to clarify the genetic cause of repeated fertilization failure after ICSI-AOA in a non-consanguineous family. Additionally, a novel compound heterozygous mutation in *WEE2* was identified.

## Case presentation

### Clinical characteristics of female proband

The infertile couple is from a non-consanguineous family. The proband is a 36-year-old woman, Chinese, Han ethnic with a 2-year history of primary infertility of unknown cause. Her husband is a 38-year-old Chinese man with a 2-year history of primary infertility; his seminal parameters showed 49% sperm motility and 4% normal sperm morphology per ejaculate. A total of three ART cycles was conducted (Table 1). During the patient’s first IVF cycle, 15 oocytes were collected; however, pronucleus formation was not observed. Late rescue ICSI was performed for 8 MII oocytes; 2 two-pronuclei-fertilized oocytes and 1 three-pronuclei-fertilized oocyte were obtained. Unfortunately, the zygote stopped developing at the 2-cell stage. During the second ICSI cycle, 12 oocytes (9 MII oocytes) were collected but none was fertilized. During the third ICSI-AOA cycle, the oocytes were dispersed in a calcium ionophore solution containing 10 μmol/L of A23187 (I9657, Sigma-Aldrich, St. Louis, MO, USA) for 10 min at 37 °C with 6% CO_2_. Totally 15 oocytes (11 MII oocytes) were collected followed by ICSI-AOA but PN formation was not observed.

**Table 1:** Clinical characteristics of the female proband

Case	Age (years)	Duration of infertility (years)	1st IVF cycle		1st r-ICSI cycle				2nd ICSI cycle		3rd ICSI-AOA cycle	
			Oocytes	2PN	MII	2PN	3PN	Available embryos	MII	2PN	MII	2PN
II-1	36	2	15	0	8	2	1	0	9	0	11	0

*MII* metaphase II, *PN* pronucleus, *r-ICSI* rescue ICSI

### Sequencing analysis

Genomic DNA was extracted from peripheral blood samples of the couple and their parents. Whole-exome capture and sequencing were performed following the standard protocols provided by BGI Genomics (BGI, Shenzhen, China) and Tianjin Medical Laboratory BGI (Tianjin, China). The genomic DNA library was subjected to Agilent 2100 Bioanalyzer and BMG and sequenced with MGISEQ-2000. The sequencing depth of WES was 100×. For Sanger sequencing, the primers and conditions used for PCR are listed in Additional file 1: Table S1. The PCR product was sequenced on an ABI 3100 DNA analyzer (Applied Biosystems, Foster City, CA, USA) and the data were analyzed with Sequencer 4.9 software.

**Fig. 1:**
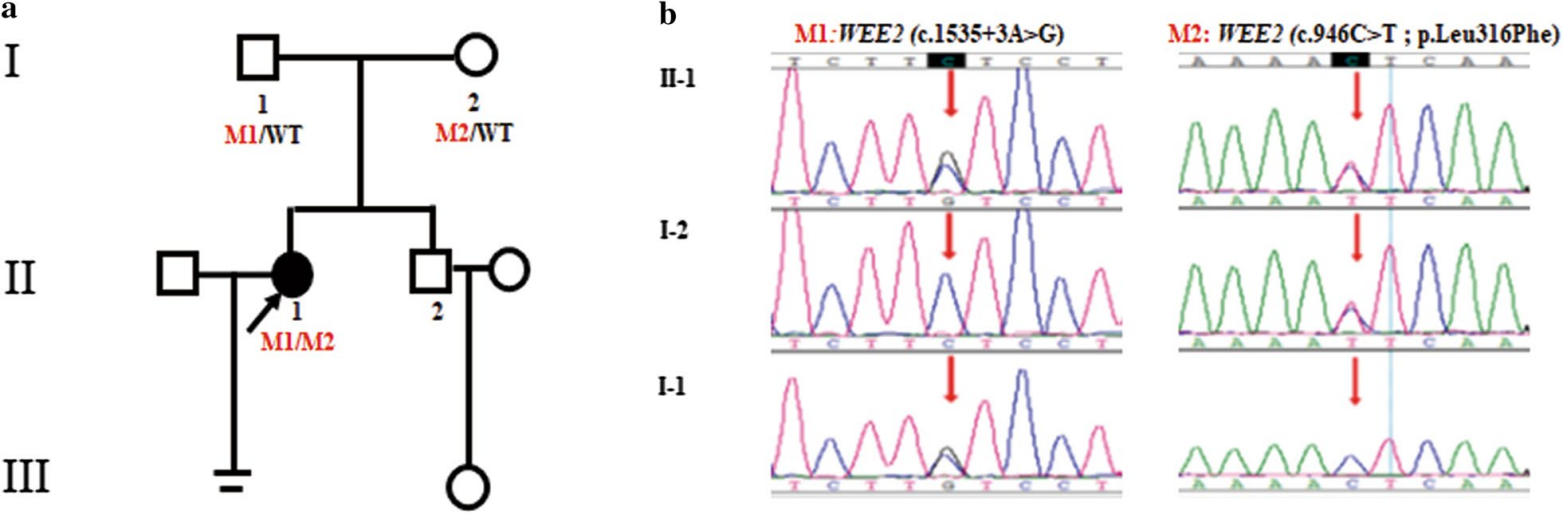
Trios analysis of woman with fertilization failure. The black circle indicates the female proband. The red arrows in the chromatograms indicate the mutation locations

**Fig. 2:**
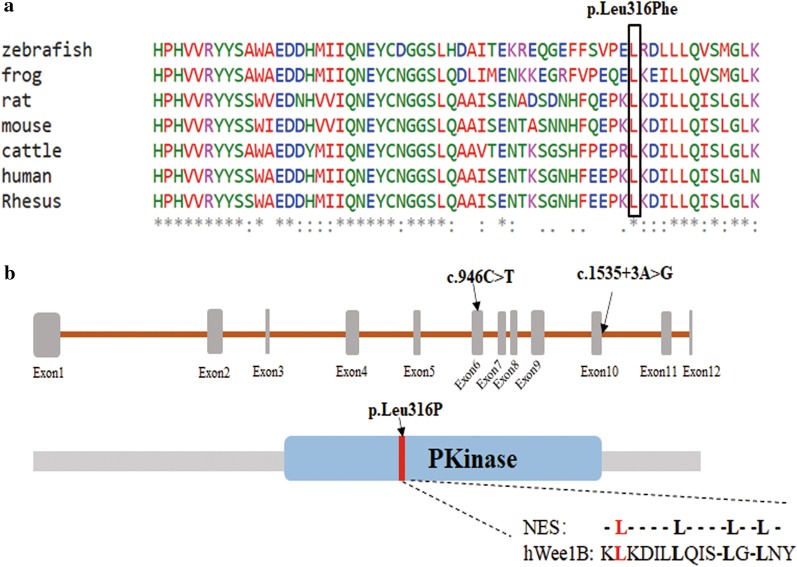
Conservation analysis and location, of *WEE* mutations. **a** Mutation p. Leu316Phe was conserved among different species. **b** Position of mutation c.946C > T (p. Leu316Phe) and c.1535 + 3A > G in the genome and protein structure. The red region represents the nuclear export sequence (NES)

Aneuploidy or pathogenic microdeletion/microduplication (> 100 Kb) was not detected in the infertile couple by low-coverage whole-genome sequencing. A novel compound heterozygous mutation c.1535 + 3A > G and c.946C > T (p. Leu316Phe) in *WEE2* was found in the infertile female proband by whole-exome sequencing. The allele frequencies of variations c.1535 + 3A > G and c.946C > T were not listed in Exome Aggregation Consortium (ExAC; exac.broadinstitute.org) or the 1000Genomes Project Database (browser.1000genomes.org) (see Table 2). The identified missense mutation c.946C > T (p. Leu316Phe) was conserved among different species assessed with Clustal Omega software (Science Foundation Ireland) (Fig. 2a). Sanger sequencing validated that variation c.1535 + 3A > G was inherited from her father and variation c.946C > T (p. Leu316Phe) was inherited from her mother. As shown in Fig. 1, Trios analysis revealed an autosomal recessive mode of the detected variations in *WEE2*.

**Table 2:** Overview and bioinformatic prediction of the novel *WEE2* mutations

Genomic position on chr. 7 (bp)	Exon	cDNA change	Protein change	Mutation type	1000 genome	ExAC	Human splicing finder	SIFT-2	PolyPhen-2	Mutation taster
141,427,249	IVS10	c.1535 + 3A > G	–	Splicing	Not found	Not found	Broken WT Donor Site	–	–	Disease causing
141,422,999	exon6	c.946C > T	p.Leu316Phe	Missense	Not found	Not found	–	Deleterious	Probably damaging	Disease causing

### Function prediction of the mutations

The locations of the mutations in *WEE2* and WEE2 protein are shown in Table 2 and Fig. 2b. Variation c.1535 + 3A > G was at an intervening sequence 3 base pairs away from the edge of exon 10. Variation c.946C > T was in exon 6 and caused an amino acid substitution at position 316 in WEE2 protein. This missense mutation was in both the PKinase domain and putative nuclear export sequence (NES) of WEE2.

The pathogenicity of the variants was assessed using four software programs: SIFT (sift.jcvi.org), PolyPhen2 (www.genetics.bwh.harvard.edu/pph2/), Mutation Taster (www.mutationtaster.org/), and Human Splicing Finder (www.umd.be/HSF3/). As shown in Table 2, variation c.1535 + 3A > G in *WEE2* was predicted to break the wild-type donor site and affect splicing. For the cryptic site used, the exon length alteration was expected to be 152 base pairs according to Human Splicing Finder software. The missense mutation p.Leu316Phe of *WEE2* was predicted to be pathogenic by SIFT, PolyPhen-2, and Mutation Taster software.

## Discussion and conclusion

In this study, through whole-exome sequencing, a novel compound heterozygous mutation in *WEE2* was identified in an infertile female who experienced repeated fertilization failure even after ICSI-AOA.

*WEE2* was previously identified as a testis-abundant gene [7] but has not been demonstrated to be associated with male infertility in humans. Since 2011, Wee2 has been considered as a crucial factor contributing to oocyte activation and pronucleus formation. In 2018, causative *WEE2* mutations of human fertilization failure were first identified in humans. Jing et al. detected mutations in *WEE2* in 20.8% (5/24) of women with TFF or poor fertilization, whereas Shuai et al. identified *WEE2* mutations in nearly 6% (5/90) women with TFF, indicating that *WEE2* mutations play important role in TFF cases. Nineteen genetic mutations have been detected in *WEE2* in women who experienced fertilization failure. We summarized all previously reported variations of *WEE2* in affected cases with total fertilization failure/poor fertilization in Table 3. Nearly all the patients with *WEE2* mutations presented with complete obstacles in PN formation on day 1 (D1) from MII oocytes despite the use of ICSI or the calcium ionophore A23187 for activation. However, cases with mutation c.1228C > T, c.1576T > G and c.293_294ins presented with low fertilization rate and none normal cleavage embryos [11, 12, 14]. In our study, we identified a novel compound heterozygous mutation in *WEE2*, expanding the mutation spectrum of this gene in fertilization failure.

**Table 3:** Variations of *WEE2* in affected cases with fertilization failure/poor fertilization

Location	Sequence variation	Amino acid change	Mutation type	Compound Het/Homo	Function	Phenotype MII 2PN		References
Exon 1	c.220_223delAAAG	p.Glu75Valfs*6	Frameshift	Homozygous	Protein degradation; Decreased pY15 of Cdc2	18	0	[6]
Exon 1	c.293_294ins	p.Pro98Pro fsX2	Frameshift	Homozygous		19	1	[12]
Exon 1/Exon 4	c.220_223delAAAG c. 598C > T	p.E75Vfs*6 p.R200X	Frameshift Nonsense	Compound heterozygous		7	0	[11]
Exon 1/Exon 5	c.341_342delAA c.864G > C	p.Lys114Asn fsX20 p.Gln288His	Frameshift Missense	Compound heterozygous		22	0	[12]
Exon 1/ Exon 8	c.220_223delAAAG c.1221G > A	p.E75Vfs*6 p.D408Vfs*1	Frameshift Splicing	Compound heterozygous	Splicing mutation resulted in a predicted truncated protein	27	0	[11]
Exon 1/Exon 9	c.1A > G c.1261G > A	p.0? p.Gly421Arg	Frameshift Missense	Compound heterozygous		25	0	[12]
Exon 3	c. 585G > C	p.Lys195Asn	Missense	Homozygous	No phosphorylated CDC2	14	0	[14]
Exon 4	c.619C > T	p. R207C	Missense	Homozygous	May breaking the hydrogen bonds with E211 and S277	40	0	[10]
Exon 4	c.700G > C	p.Asp234His	Missense	Homozygous	Decreased WEE2 protein; Decreased tyrosine 15 phosphorylation (pY15) of Cdc2	3	0	[6]
Exon 4/Exon 6	c. 725G > C c. 997 T > C	p.R242P p.S333P	Missense Missense	Compound heterozygous		4	0	[11]
Exon 4/ Exon 9	c.598C > T c.1319G > C	p.Arg200Ter p.Trp440Ser	Missense Missense	Compound heterozygous	Truncated protein Abnormal subcellular localization and reduced WEE2	37	0	[13]
Exon 6	c.1006_1007insTA	p.His337Tyrfs*24	Frameshift	Homozygous	Protein degradation; Decreased pY15 of Cdc2	64	0	[6, 14]
Exon 6/IVS 7	c.1006_1007dup c.1136-2A > G	p.His337Tyrfs*24 p.Gly379Glufs*6/p Asp380Leufs*39	Frameshift Splicing	Compound heterozygous	Decreased WEE2 protein; No phosphorylated CDC2	19	0	[14]
Exon 6/Exon 9	c.991C > A/c.1304_1307delCCAA	p.His331Asn p.Thr435Met fsX31	Missense Frameshift	Compound heterozygous		27	0	[12]
Exon 6/Exon 9	c.1006_1007insTA c.1286_1288delGAG	p.H337Yfs*24 p.G429del	Frameshift Deletion	Compound heterozygous		42	0	[11]
Exon 8	c. 1184G > A	p.G395E	Missense	Homozygous		18	0	[11]
Exon 9	c.1228C > T	p.Arg410Trp	Missense	Homozygous		71	6	[11, 14]
Exon 10	c.1473dupA	p.Thr493Asnfs*39	Frameshift	Homozygous	Decreased WEE2 protein; Decreased pY15 of Cdc2	8	0	[6]
Exon11	c.1576T > G	p.Tyr526Asp	Missense	Homozygous		27	1	[12]

*MII* total fertilization failure metaphase II, *PN* pronuclei

The compound heterozygous mutation c.1535 + 3A > G and c.946C > T (p. Leu316Phe) follow an autosomal recessive pattern and may contribute to the loss of WEE2 protein function. Variant p. Leu316Phe is in the PKinase domain of WEE2 protein, which has protein kinase activity [15]. A mutation in the PKinase domain may affect the serine phosphorylation of WEE2, resulting in impairment of Cdc2 phosphorylation and leading to the failure of fertilization [6]. In addition, variant p. Leu316Phe is in the NES of WEE2. Jeong et al. found that nucleocytoplasmic shuttling of Wee2 in mouse was mediated by NES sequences [16]. Thus, mutation in this region may disrupt the nuclear localization of WEE2.Variation c.1535 + 3A > G is likely affects splicing. This splicing mutation may result in a 152-base pair alteration in exon length and generate a truncated protein.

In this study, ICSI-AOA via chemical activation of A23187 was performed in a patient after two failed IVF/ICSI cycle. Unfortunately, fertilization failure was encountered in ICSI-AOA cycle. Consistent with our observations, early Ca^2+^ signaling during egg activation was previously found to be normal in *Wee2* knockdown mouse [9]. We predicted that Ca^2+^ release from the oocyte endoplasmic reticulum and calcium-calmodulin–dependent kinase II (CaMKII) activation occurred normally in this patient. However, CaMKII-triggered WEE2 phosphorylation, inhibitory CDC2 phosphorylation, and maturation-promoting factor destruction were impaired [17]. Receiving the donated egg is an option for patients with defective WEE2. In addition, Sang et al. injected WEE2 complementary RNA into the oocyte of a patient with defective *WEE2* and found that fertilization failure was rescued and blastocysts were formed in vitro. Further studies are required to develop treatments for patients with *WEE2* defects.

In conclusion, we identified a novel compound heterozygous mutation in *WEE2* responsible for fertilization failure. Our results expand the spectrum of *WEE2* mutations and provide genetic evidence for fertilization failure.

## Supplementary information

**Additional file 1: Table S1.** PCR primers and conditions used for Sanger sequencing. Primer pairs and the size of PCR products were shown. F, forward; R, reverse.

## Data Availability

All datasets generated for this study are included in the article.

## Abbreviations

TFF: Total fertilization failure
IVF: In vitro fertilization
ICSI: Intracytoplasmic sperm injection
AOA: Artificial oocyte activation
ICSI-AOA: ICSI followed by AOA
