# Supplementary material for: Novel compound heterozygous mutation in WEE2 is associated with fertilization failure: case report of an infertile woman and literature review
Source: BMC Womens Health. 2020 Nov 4;20:246. doi: 10.1186/s12905-020-01111-5 (PMC7643268; doi:10.1186/s12905-020-01111-5)
Supplement: Supplementary file 1 — Additional file 1: Table S1. PCR primers and conditions used for Sanger sequencing. Primer pairs and the size of PCR products were shown. F, forward; R, reverse. [file 12905_2020_1111_MOESM1_ESM.docx]

Table S1 PCR primers and conditions used for Sanger sequencing

| Variation | Location | Primer Pairs | PCR size(bp) |
| --- | --- | --- | --- |
| c.1535+3A>G | IVS10 of *WEE2* | F: CCTCTTGGTCCTATATCATCAG  R: CTCAAGCAACCTAATCACAGAG | 367 |
| c.946C>T | Exon6 of *WEE2* | F: GGGCATTTGGGGCTTTGATATC  R: CAAACACCGTCCTTGACACTTG | 384 |

The PCR conditions were as follows: initial denaturation at 95°C for 5 min; then denaturation at 94℃ for 30 s, annealing at 58℃ for 30 s, and elongation at 72℃ for 30 s, repeated for 35 cycles; finally, elongation at 72℃ for 5 min.

F, forward; R, reverse.
